# Supplementary material for: Stakeholders perspective of, and experience with contact tracing for COVID-19 in Ghana: A qualitative study among contact tracers, supervisors, and contacts
Source: PLoS One. 2021 Feb 11;16(2):e0247038. doi: 10.1371/journal.pone.0247038 (PMC7877738; doi:10.1371/journal.pone.0247038)
Supplement: S1 File — (DOCX) [file pone.0247038.s001.docx]

**KEY INFORMANT INTERVIEW GUIDE**

I am going to ask you some questions about your contact tracing process, take time in answering them and feel free to ask me to explain further if the question is not clear to you. You can skip the question and return to it if you want to. If you do not want to comment on a question please say so. Please be assured that there are no wrong answers, so give me your honest response. Remember that whatever you share with me will not be identified with you but may be used as a piece of valuable information in the study

**SECTION A: DEMOGRAPHIC DATA**

1. Participant‘s Label/ Pseudonym

2. Age........................................................................................................

3. Gender ………………………………………………………………….........

4. Educational level ……………………………………………………...........

5. Profession …………………………………………………………..............

6. Marital status ……………………………………………………………....

7. Religious affiliation …………………………………………………..........

**SECTION B: GUIDING QUESTIONS**

8. Can you please tell me about yourself?

10. What is your role in the control of COVID-19?

9. What a typical day is like?

11. How is contact tracing being implemented?

12. What is the administrative structure of contact tracing?

13. What are the merits of this structure?

14. What are the demerits of this structure?

15. What is the model of contact tracing implemented in Ghana?

-What criteria do use to determine who qualifies as a contact?

16. Why are you using this specific model of contact tracing?

-How do we get contacts?

-Are there more efficient models?

17. What are the forms of contact tracing implemented in Ghana?

-How do you get cases?

18. What are the benefits of contact tracing in the control of COVID-19 generally?

19. What are the challenges facing this process?

-resources

-politics

-bureaucracy

-preparedness

20. How is the team working around them?

21. What recommendations would you give to improve contact tracing activities in Ghana

22. According to you, how is the overall implementation going so far

23. How were the communities involved?

24. What enables the smooth flow of the activities?

25. What are some of the challenges faced by your field team?

26. What is your perception of the whole process?

27. What can you attribute your success to?

2.8 What are the most needed resources for the success of the activities?

-Are they readily available?

-Where are how do you receive resources to carry out your role.

2.9 How was recruiting of contact tracers done?

-qualifications?

-previous experience

-willingness

30. Describe all the factors that affect how you are able to do your job to your satisfaction

**RECOMMENDATIONS**

31. What advice would you give a person intending to be a contact tracer?

32. How has this interview been to you?

33. Is there anything else you would like to tell me about which you think would be important for me to know?

**Closing** I am grateful for the time you have spent with me and the contribution you have made to the study. If you think now or in the next few days that our discussion has brought up things that need to be talked about please call me. I would be happy to send you the result of the study if you request it. Thank you very much.
