# Supplementary material for: Stakeholders perspective of, and experience with contact tracing for COVID-19 in Ghana: A qualitative study among contact tracers, supervisors, and contacts
Source: PLoS One. 2021 Feb 11;16(2):e0247038. doi: 10.1371/journal.pone.0247038 (PMC7877738; doi:10.1371/journal.pone.0247038)
Supplement: S2 File — (DOCX) [file pone.0247038.s002.docx]

**INTERVIEW GUIDE FOR CONTACTS**

I am going to ask you some questions about your Quarantine experience, take time in answering them and feel free to ask me to explain further if any of the question(s) is/are not clear to you. You can skip the question and return to it if you want to. If you do not want to comment on a question please say so. Please be assured that there are no wrong answers, so give me your honest response. Remember that whatever you share with me will not be identified with you but may be used as a piece of valuable information in the study

**SECTION A: DEMOGRAPHIC INFORMATION**

1. Participant ‘s Label/ Pseudonym

2. How old are you?

3. Gender

4. What is your level of education?

5. What is your profession?

6. What is your nationality?

7. Where do you live?

8. What is your marital status?

9. Which Religious faith do you belong to?

10. Where do you stay?

11. Do you currently stay with your family?

**SECTION B: GUIDING QUESTION**

**Pre-quarantine perception of risk**

Can you please share with me you view/perception and COVID-19 prior to your quarantine

-views about risk

-compliance to social distancing

-Compliance to hand hygiene

**History of exposure**

12. Can you share with me the circumstance leading to your exposure to someone with covid-19?

-where contact occurred

-first contact

-voluntary or mandatory

Duration of interaction

13. How were/are quarantine?

-by choice or not?

-alone or in a group per room?

Self-quarantine at home

**Experience during quarantine**

Can you share with me all that happen on the first day you were informed about the need for quarantine

-How information was disclosed and by whom

-counselling

-Informing family members

-provision of resources

15. Narrate a typical day in quarantine

16. How often were you visited?

**Contact tracing**

How are you reached by the health worker assigned to you

-personal face-to-face visit

-phone

How frequently are you contacted by the health workers?

17. Can you share with me what happens when you are visited/contacted on phone by the health workers t? (checking of temperature, health education, counsellimg, provision of resources

18. How are/were you treated by the routing visitors/ medical personnel?

-how did that make you feel

-did they ever make you feel uncomfortable?

-reassuring?

22. How do you feel about the government making that process compulsory for you and everyone else in your condition?

23. Did you help the medical personnel find more contacts like you?

-if yes or no why.

24. How do you feel about being quarantined?

-Did you give them ideas of how to approach?

-How did you feel being under quarantine?

**Testing and disclosure of results**

26. At the end of the contact period where u tested for covid-19?

-did you maintain the quarantine for that prescribed period.

27. How easy was it for you to go back to normal after the quarantine?

-How did the quarantine affect you in your normal life?

**Perceived effectiveness of contact tracing model**

28. How effective has this contact tracing exercise been?

29. Any suggestions to how best this would have been done?

-any recommendations to better the experience for the others still undergoing quarantine?

- What advice would you give a contact tracer?

30. Rate the whole process

31. How has this interview been to you?

32. is there anything else you would like to tell me about which you think would be important for me to know?

**Closing** I am grateful for the time you have spent with me and the contribution you have made to the study. If you think now or in the next few days that our discussion has brought up things that need to be talked about please call me. I would be happy to send you the result of the study if you request for it. Thank you very much.
