# Supplementary material for: Stakeholders perspective of, and experience with contact tracing for COVID-19 in Ghana: A qualitative study among contact tracers, supervisors, and contacts
Source: PLoS One. 2021 Feb 11;16(2):e0247038. doi: 10.1371/journal.pone.0247038 (PMC7877738; doi:10.1371/journal.pone.0247038)
Supplement: S3 File — (DOCX) [file pone.0247038.s003.docx]

**IN-DEPTH INTERVIEW GUIDE FOR CONTACT TRACERS**

I am going to ask you some questions about your contact tracing experiences, take time in answering them and feel free to ask me to explain further if the question is not clear to you. You can skip the question and return to it if you want to. If you do not want to comment on a question please say so. Please be assured that there are no wrong answers, so give me your honest response. Remember that whatever you share with me will not be identified with you but may be used as a piece of valuable information in the study

**SECTION A: DEMOGRAPHIC INFORMATION**

1. Participant‘s Label/ Pseudonym

2. How old are you?

3. Gender

4. What is your level of education?

5. What is your profession?

Which year did you obtain the professional qualification

6. What is your nationality?

7. Where do you live?

8. What is your marital status?

9. Which Religious faith do you belong to?

10. Where do you stay?

11. Do you currently stay with your family?

**SECTION B: GUIDING QUESTION**

**1. KNOWLEDGE OF CONTACT TRACER ABOUT COVID-19**

1.1 What is COVID-19

-transmission

-treatment

-current status

1.2 What made you decide to be a contact tracer?

-what motivates you?

-What kept you going as a contact tracer?

1.3 Can yo share with us how you were selected as a contact tracer?

-pevious experience in contact tracing

-training before deployment

What is your opinion about you level of preparedness to do the job?

-IT

- Skills

-knowledge

**2. CONTACT TRACER EXPERIENCE**

2.1 What is your typical day like?

- Do you have a schedule of checking up on your contacts?

-Do you believe/think the contacts in their homes observed the curfew… why not?

2.2 What is your role as a contact tracer in the control of COVID-19?

2.3 Generally what was the experience of your first visit to each contact?

-Hostile

-welcoming

-informed

-grateful

2.4 What was your perception of the contacts psychological wellbeing during the follow-up period

-stress

2.5 How did you feel when some of the assigned contacts to you turned out positive COVID-19?

2.6 What have you learned from this experience?

2.7 Have you been a contact tracer before?

-What differences have you encountered?

2.8 Do you think you have achieved your intended goal as a contact tracer?

-if not what hindered your success?

-What do you attribute your success to, thus far?

2.9 What’s the weirdest, most shocking thing found in the field?

2.10 How has being contact tracer affected your life?

-Did the job affect how you interacted with your family/ close contacts back home?

- How has being a contact tracer altered your choice of activities you participate in?

**3.0 CONTACT TRACING PROCESS**

3.1 What is your view about Ghana’s preparation to contain COVID-19 spread?

What are you opinion about the interventions being implemented in control COVID-19?

3.2 What is the overview of contact tracing activity in Ghana as a control mechanism?

- Who and how are your duties assigned to you?

-Who recruited you?

-Who do you report to?

-What is the reporting structure like?

3.3 What are your views of the contact tracing process?

3.4 How best would you implement the whole process?

-What would you do differently if you were the lead in surveillance and contact tracing

**4.0 CONTACT TRACER CHALLENGES AND BENEFITS**

4.1 Tell me about the difficulties you face

-What demotivated you the most?

-How did you overcome them? how do you deal with the difficulties you face?

-How have rumors impacted the process?

4.2 Tell me about the resources needed?

-Where are how do you receive resources to carry out your role.

4.3 Describe all the factors that affect how you perform at your job

-are you proud to be a contact tracer?

**5.0 RECOMMENDATIONS**

5.1 What advice would you give a person intending to be a contact tracer?

5.2 How has this interview been to you?

5.3 Is there anything else you would like to tell me about which you think would be important for me to know?

**Closing** I am grateful for the time you have spent with me and the contribution you have made to the study. If you think now or in the next few days that our discussion has brought up things that need to be talked about please call me. I would be happy to send you the result of the study if you request for it. Thank you very much.
