# Supplementary material for: Stakeholders perspective of, and experience with contact tracing for COVID-19 in Ghana: A qualitative study among contact tracers, supervisors, and contacts
Source: PLoS One. 2021 Feb 11;16(2):e0247038. doi: 10.1371/journal.pone.0247038 (PMC7877738; doi:10.1371/journal.pone.0247038)
Supplement: Attachment — Submitted filename: Review_Plos_18_09.docx [file pone.0247038.s004.docx]

**Review summary**

The main objective of this qualitative study is to document the experiences of diverse stakeholders (contact tracers, supervisors, and contacts) when contact tracing activities began in Ghana following the outbreak of SARS CoV-2 in March 2020. The study claims to follow a phenomenology and narrative approach. Authors described contact tracers’ activities including training and deployment and described some of the difficulties encountered by contacts of confirmed cases who had to self-quarantine. Lack of PPE and remuneration were major issues for contact tracers. Delays in receiving results and poor coordination of the process also prevented contact tracing activities to be effective.

This paper shows important issues that for both sides of the story, which shed light on the difficulties people experience when there is an outbreak of an unknown virus. There is little evidence about these experiences and there are some important findings in this paper. For instance, it is important to highlight the lack of PPE and remuneration for people who are crucial in fighting the virus. However, I feel that the manuscript could be improved if some of the below comments are considered.

The manuscript will also benefit from proofreading, as there are several sentences that need revising.

**Abstract**

Lines 21-22: Needs to specify that COVID-19 is caused by SARS CoV-2

Lines 24: replace comma with ‘and’ (contact tracers and their supervisors)

Lines 26: N=27 instead of (27)

Lines 30: Delete ‘Then’

Line 32-33: Sentence is incomplete, needs revising

Lines 35: contacts (add an s)

Line 37: Use past tense (was instead of is)

Line 39: Delete ‘In addition to this’

Lines 43: Delete ‘The study concludes that the’

Line 46: needs (add an s)

**Background**

Line 56: “A study from China” is repetitive, change the sentence so it flows better.

Line 62: I thought the WHO declared a pandemic on 12^th^ March 2020 –please check this fact.

Line 64: add comma (spread and severity, and by the alarming…)

Line 64: It was not evident in June, it was evident before that. Please rewrite this sentence.

Line 73: ‘is’ transmitted instead of ‘was’ transmitted. What do you mean by contacts in that sentence, contact with infected surfaces?

Line 74: R_0_ has been estimated

Line 79: cases have continued to rise instead of using ‘suddenly’ (it didn’t happen from one day to the other)

Line 80: needs references, what is the source of this statistics?

Line 83: Needs reference of EPRP after the quote.

Line 89: for the containment phase of COVID-19 ‘was’ contact tracing

**Study Design**

Line 174: In all, 27 people

Line 176: the table needs a title

**Data Collection Tool**

Line 202: The data was collected between 6th and 26^th^ April 2020

Line 207: Sentence needs revising

**Results**

Data in Table 2 could be summarised in a better way. Socio-demographic characteristics could be included in the first column while category of interviewees (supervisors, contact tracers, contacts) could be in 3 separate rows. Age can be given as the average (±), then frequencies (%) could be calculated for sex, religion, marital status, professional qualification categories can be created or educational level could be reported instead).

Line 313: remine?

Data presented in pages 13-18 (until line 352) is relevant because it is important for readers to know what other countries have done. I had not heard of any countries doing the 2km radius, which I found very interesting, but the quotes in these pages seem to repeat what is already written in the text.

For instance, it would be more interesting to know if there were any discrepancies in the way contact tracers defined a contact than just adding a quote. If the intention is to say that the contact tracers really understood what a ‘contact’ is, then that is what needs to be included in the narrative.

In this section, I would like to know more about: Did contact tracers feel they had the knowledge and confidence to do the job? Did they complained about the hours worked? How difficult it was to cover the 2km radius?

Other example, lines 259-266, there is a discrepancy between what the monitoring guideline says and what contact tracers do (e.g. once a day instead of twice). Authors could do a better job by building that narrative instead of just repeating what the participants said in the interview.

From line 352, the data flows in a better way and quotes actually provide something extra that is not included in the text.

Were supervisors aware of the lack of PPE? Do you have any quotes from them that illustrates that they were aware of the importance and the risks of not providing PPE? Or maybe they thought it wasn’t necessary.

**Discussion**

Lines 472-477: Apart from the 1^st^ sentence, the rest should be deleted. That information belongs to the methodology section.

Line 495: change to COVID-19

The issue of lack of PPE and remuneration was not discussed in here and I wonder if there is evidence of this in other outbreaks, particularly Ebola. What were the experiences of contact tracers during the Ebola outbreak in relation to PPE, training, etc? There are only a few studies about contact tracing but there must be some evidence to make some comparisons.

The lack of PPE is a very important point to discuss to ensure that the job is safe and that contact tracers are treated fairly as key workers (see below comment). In other countries contact tracers do it remotely but visiting houses and having face-to-face contact with possible cases puts them at risk. On the other hand, is there any evidence that full PPE is needed (line 418)? It is possible that contact tracers also need to be trained on the risk of infection when doing contact tracing and taking samples.

What are the strengths and limitations of the study?

**Conclusions**

Important challenges such as PPE and remuneration need to be discussed in the discussion section to be able to conclude “Lastly, the issue of protection and motivation of health workers need to be taken seriously at all levels”.
